# The Effects of Post-Surgical Pregnancy on Weight Loss Trajectories after Bariatric Surgery: Are Initial Weight and Age Prognostic Factors? †

**DOI:** 10.3390/jcm13051264

**Published:** 2024-02-23

**Authors:** Juan S. Barajas-Gamboa, Mohammed Sakib Ihsan Khan, Jerry T. Dang, Gustavo Romero-Velez, Gabriel Diaz Del Gobbo, Mohammed Abdallah, Juan Pablo Pantoja, Carlos Abril, Alfredo D. Guerron, Terrence Lee-St. John, Ricard Corcelles, John Rodriguez, Matthew Kroh, M. Jean Uy-Kroh

**Affiliations:** 1 Digestive Disease Institute, Cleveland Clinic Abu Dhabi, Abu Dhabi 112412, United Arab Emirates; barajaj@clevelandclinicabudhabi.ae (J.S.B.-G.); mohammed.khan@ku.ac.ae (M.S.I.K.); gabodelgobbo@hotmail.com (G.D.D.G.); abdallm2@clevelandclinicabudhabi.ae (M.A.); pantojj@clevelandclinicabudhabi.ae (J.P.P.); abrilc@clevelandclinicabudhabi.ae (C.A.); guerrod@clevelandclinicabudhabi.ae (A.D.G.); leestjt@clevelandclinicabudhabi.ae (T.L.-S.J.); rodrigj2@clevelandclinicabudhabi.ae (J.R.); 2 Digestive Disease & Surgery Institute, Cleveland Clinic, Cleveland, OH 44195, USA; romerog4@ccf.org (G.R.-V.); corcelr@ccf.org (R.C.); krohm@ccf.org (M.K.); 3 Cleveland Clinic Lerner College of Medicine, Case Western Reserve University, Cleveland, OH 44106, USA; 4 Women’s Health Institute, Cleveland Clinic, Cleveland, OH 44195, USA

**Keywords:** bariatric surgery, pregnancy, weight loss, weight regain, sleeve gastrectomy, Roux-en-Y gastric bypass

## Abstract

**Introduction:** A substantial percentage of patients undergoing bariatric surgery are of childbearing age. Pregnancy outcomes after bariatric surgery are known. However, there are limited data on the impact of pregnancy on weight loss after surgery. **Objectives:** This study aims to evaluate the effects of pregnancy on post-bariatric surgery weight loss trajectories (WLTs) and to determine the association with age and initial weight. **Methods:** All who had primary bariatric surgeries (Roux-en-Y gastric bypass or sleeve) between September 2015 and July 2020 were classified into two groups: post-surgery gravid (GG) and post-surgery non-gravid (NG). WLTs were examined using a random intercept mixed-effects model with repeated measures nested within patients. The post-surgery/pre-gravid time phase (PoPG) was modelled using a third-degree polynomial. For GG, two third-degree spline functions modelled the post-surgery while gravid (PoWG) and post-partum (PoPP) time phases. Age and initial weight were used to control for pre-existing differences during PoPG. Weight differences at 6 months PoPP were examined by applying general linear hypothesis testing to the mixed-model results. **Results:** A total of 508 patients were included, 20 in GG and 488 in NG. The mean age at surgery was 33 years in GG and 37 years in NG. The mean initial BMI was 47 kg/m^2^ and 43 kg/m^2^, respectively. During PoPG, adjusted average weight in both groups follows the path across time. For GG, weight decreases and then increases during PoWG. For GG during PoPP, weight immediately decreases after delivery and then increases over time to levels similar to NG. Weight differences at 6 months PoPP for GG and NG were not statistically different. Older age was associated with reduced weight loss during PoPG by Baseline Age, while higher initial weight was associated with increased weight loss during PoPG by Baseline Weight. In both instances, these effects attenuate over time. **Conclusions:** This model indicates that pregnancy following bariatric surgery affects WLT during PoWG and PoPP, and no difference in weight is expected after 6 months post-gravid. Age and initial weight could be considered prognostic factors during PoPG. Patients wishing to conceive should undergo preconception counselling and be advised to avoid pregnancy during the period of rapid weight loss. They also should be informed that WLT may vary during pregnancy and early post-partum.

## 1. Introduction

Over the last four decades, the prevalence of obesity—conventionally defined as body mass index (BMI) ≥ 30 kg/m^2^—has progressively increased to pandemic proportions [1]. Obesity is associated with a range of non-overlapping diseases, including digestive, respiratory, neurological, musculoskeletal, and infectious disorders, as well as cardiometabolic conditions such as coronary heart disease (CHD), stroke, or diabetes mellitus [1]. Although lifestyle modifications (diet and exercise) and anti-obesity pharmacotherapy are recommended as the main treatment approach for obesity, most individuals find it hard to implement it [2,3]. Hence, bariatric surgery remains the most clinically effective and cost-effective intervention for people with morbid obesity [2]. The adoption of bariatric surgery worldwide has increased exponentially in the last decade [2], including for women of childbearing age. Thus, it is common to encounter pregnancy following bariatric surgery.

The most weight loss occurs within the first 12–18 months post-surgery [4]. As such, several societies, such as the American Association of Clinical Endocrinology, the Obesity Society, and the American Society for Metabolic and Bariatric Surgery, recommend that pregnancy should be avoided for 12–18 months after bariatric surgery [4]. At the same time, the American College of Obstetricians and Gynaecologists proposed a longer time period of 12 to 24 months post-surgery [4,5]. The duration proposed is based on the theory that fetal development during a period of maternal malnutrition could lead to adverse maternal and fetal consequences [6]. These consequences may include severe maternal nutritional deficiencies, pregnancy complications, low fetal birth weight, and congenital fetal malformations [7,8]. However, several studies have shown no significant difference in perinatal outcomes between pregnancies conceived early (within 12 months post-surgery) compared to late (after 12 or 18 months post-surgery) [6]. Furthermore, this delay imposes unnecessary restrictions on family planning and patients with infertility issues, advanced maternal age, and other age-related comorbidities [6].

Pregnancy after bariatric surgery offers several benefits for women with severe obesity, particularly in terms of improved fertility and reduced risks of certain pregnancy complications. A retrospective study by Teitelman et al. involving 195 female patients showed that bariatric surgery can enhance fertility by resolving anovulation and by restoring normal menstrual cycles [9]. Additionally, women who conceive after bariatric surgery have lower rates of gestational diabetes, hypertensive disorders in pregnancy, preeclampsia, and chronic hypertension compared to those who have not undergone the surgery [6]. These findings suggest that pregnancy following bariatric surgery can improve maternal and fetal health outcomes [10,11,12].

Only a few studies have investigated gestational weight gain [4,13,14,15], but to our knowledge, none of the studies have evaluated the weight loss trajectories (WLTs) for pre-gravid, gravid, and post-partum females after bariatric surgery. Hence, this study aims to evaluate the effects of pregnancy on post-bariatric surgery WLTs and to determine the association with age and initial weight.

## 2. Materials and Methods

**Figure 1:**
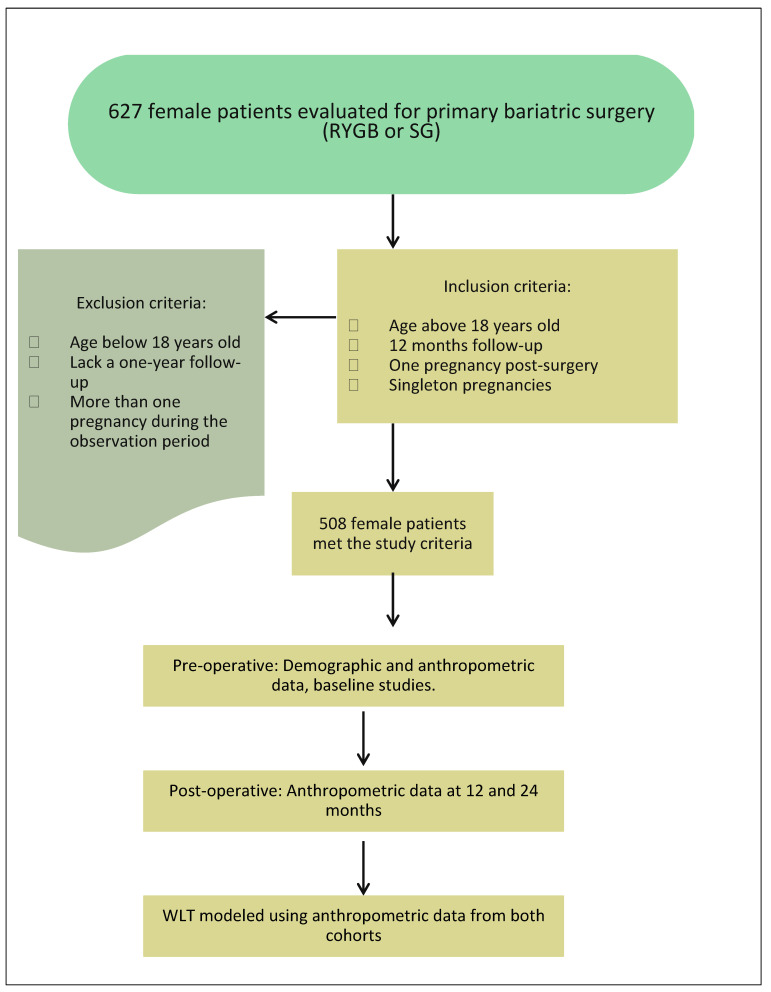
Study flow chart.

Study design and ethical approvals: This was a retrospective study conducted with the approval of the Institutional Review Board (IRB). The approval number was A-2017-029 (Figure 1).

Objective: to evaluate the effects of pregnancy on post-bariatric surgery WLTs and to determine the association with age and initial weight.

Population: Female patients who underwent primary bariatric surgery from September 2015 to July 2020. The patients were divided into two groups: 1. post-surgery gravid group (GG) and 2. post-surgery non-gravid group (NG).

### 2.1. Selection Criteria

Inclusion criteria: These criteria included individuals more than 18 years old and above who underwent primary Roux-en-Y gastric bypass (RYGB) and sleeve gastrectomy (SG) procedures. Additionally, participants were required to have a minimum of 12 months post-surgery follow-up. Patients in the GG required pregnancy during the post-surgery observation period. Patients in the GG included only singleton pregnancies and 37 weeks and greater deliveries.

Exclusion criteria: This included patients less than 18 years old, those who underwent revisional bariatric surgery, and patients who lacked a one-year follow-up post-surgery. Additionally, individuals who experienced more than one pregnancy during the observation period were excluded from the study.

### 2.2. Pre-Operative Management

The pre-operative assessment included a comprehensive evaluation by our integrated team specializing in primary metabolic surgical interventions. The pre-operative diagnostic measures comprised an esophagogastroduodenoscopy (EGD), a radiographic study of the upper gastrointestinal tract with contrast enhancement, and extensive blood chemistry profiling. Acquisition of abdominal computed tomography (CT) imaging and abdominal sonographic scans was selectively performed based on the clinical judgment of the attending medical professionals.

### 2.3. Surgical Techniques and Bariatric Procedures

SG: The patient was transported to the operating suite and verified via their full name, medical record identification, and birth date. A pre-operative team meeting, including the anesthesiology and surgical groups, was conducted. The patient was positioned horizontally on the surgical table. The abdominal area was sanitized and shrouded following standard aseptic protocols. A surgical pause for final verification was observed. Entry into the peritoneal space was made using a 5 mm viewing trocar situated in the upper left abdominal quadrant. A pneumoperitoneum was created. Local anesthesia was applied bilaterally in the transversus abdominis plane. Trocar insertion followed a smooth U-shaped configuration.

A Nathanson liver retractor was applied under visual guidance. Dissection commenced with the removal of the phrenoesophageal adipose pad, revealing the angle of His. The greater curvature was freed by severing the gastrocolic ligament, proceeding distally and halting approximately 5 cm before the pyloric valve. The posterior short gastric vessels were severed to fully free the upper stomach section. Using a 40 French Bougie in place, the gastric sleeve was fashioned using successive applications of the gastrointestinal anastomosis (GIA) stapling device, reinforced by staples. Blood control was confirmed to be excellent. The excised tissue was extracted, and the entry site was sutured using a #0 Vicryl in a figure-eight configuration. An endoscope was inserted orally and guided visually to the duodenum to ensure the gastric sleeve was unobstructed and leak-free. Following the removal of the retractor, the pneumoperitoneum was released. The incisions were sutured using 4-0 Monocryl. The patient showed good tolerance to the procedure and was subsequently moved to the post-anesthesia care unit in stable condition.

RYGB: The procedure was initiated by transferring the patient to the operating area, where they were identified by their full name, medical record identifier, and date of birth. A pre-operative gathering involving the anesthesiology and surgery teams was conducted. The patient was laid horizontally on the operating platform, and the abdominal region was sterilized and covered as per the standard aseptic technique. A pause for a final procedural confirmation was observed. Access to the peritoneal cavity was gained via a 5 mm optical trocar placed in the left upper quadrant, establishing pneumoperitoneum. Bilateral TAP blocks were administered. Trocars were situated following a gentle U-pattern and a Nathanson liver retractor was employed under visual guidance.

The surgical team proceeded to locate the left gastric pedicle and transect the descending branch just distal to it using a reinforced purple load. A series of endo-GIA stapler firings were employed to construct a diminutive gastric pouch. Subsequently, the ligament of Treitz was located, and 100 cm distal to this landmark, the bowel was transected with a single application of the GIA stapler utilizing a tan load. Additional mesentery was dissected using an ultrasonic dissector. A Roux limb measuring 100 cm was prepared, and a side-to-side jejunojejunostomy was established with a single firing of the 60 mm GIA stapler, also with a tan load. The enterotomy was closed with a continuous 2-0 Vicryl suture.

Closure of the mesenteric defect was accomplished using 2-0 Ethibond. An omental division was executed. The team then created the gastrojejunostomy using a linear stapler and secured the Pseudo-Petersen’s defect with a continuous 2-0 Ethibond suture. Closure of all 12 mm trocar sites was achieved with a #0 Vicryl using the Carter–Thomason device. To assess the patency of the anastomosis, the bowel was clamped, and a front-viewing endoscope was introduced orally under direct vision, navigating through to the jejunum. The anastomosis was confirmed to be broadly unobstructed, with no signs of intraluminal hemorrhage or leakage. The retractor was removed, and the pneumoperitoneum was discharged. Incisions were sutured using 4-0 Monocryl. The patient demonstrated good procedural endurance and was subsequently moved to the post-anesthesia care unit in a stable condition.

### 2.4. Post-Operative Management

Following the operation, patients were transferred to the surgical unit, where they were managed according to a regimented post-operative recovery plan. This protocol included initiating early movement, employing a multimodal approach to minimize the use of narcotics for pain relief, and beginning a clear fluid diet on the day after the surgery. Post-operative observation was maintained for potential complications. Discharge criteria included the patient’s ability to consume sufficient amounts of fluids by mouth. Follow-up care was scheduled at our outpatient facility, with visits at one week and one-month post-surgery, followed by biannual assessments, all conducted by our comprehensive care team.

### 2.5. Research Procedures and Data Collection

Pre-operative data were collected for patients who underwent primary bariatric SG and RYGB surgery, which included anthropometric data (weight and height), comorbidities, and blood chemistry data. Additional data were collected for diabetes mellitus patients including insulin use and medication use. Intraoperative findings and post-operative complications were also collected. Anthropometric data were collected at 12 months and 24 months post-operatively. All the data was collected from electronic medical records. The changes in the BMI and weight were analyzed between GG and NG.

**Figure 2:**
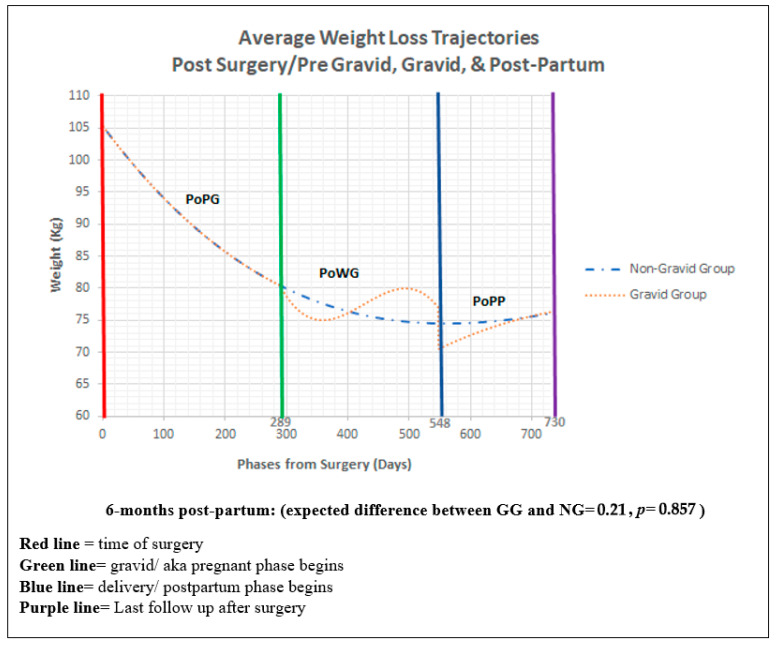
Average weight loss trajectories. Abbreviations: PoPG: post-surgery/pre-gravid; PoWG: post-surgery while gravid; PoPP: post-surgery post-partum.

Data Analysis: WLTs were examined using a random intercept mixed-effects model with repeated measures nested within patients (Figure 2). The post-surgery/pre-gravid time phase (PoPG) was modelled using a third-degree polynomial. For GG, two third-degree spline functions modelled the post-surgery while gravid (PoWG) and post-partum (PoPP) time phases. Age and initial weight were used to control for pre-existing differences during PoPG. Weight differences at 6 months PoPP were examined by applying general linear hypothesis testing to the mixed-model results.

### 2.6. Definitions

PoPG: post-surgery/pre-gravid time phase is the period between undergoing the initial bariatric surgery and conceiving a child.

PoWG: post-surgery while gravid is the period preceding the delivery following the bariatric surgery while being pregnant.

PoPP: post-surgery post-partum is the period after child delivery following the pregnancy after bariatric surgery.

### 2.7. Study Limitations

This retrospective analysis, while based on data prospectively collected from patients, comes with inherent restrictions that warrant consideration. Case selection by the authors carries the potential for selection bias due to the non-random nature of the process, which might lead to the preferential selection or omission of certain patient groups, thus skewing the outcomes. Additionally, the dependence on the completeness of existing medical records introduces the possibility of data gaps, which could affect the study’s accuracy and the integrity of its conclusions. Importantly, the Patients’ Healthy Eating Index (HEI) scores were not known, omitting a crucial aspect of nutritional status that could significantly influence health outcomes. Furthermore, an imbalance in group sizes was present, with 20 patients in the GG and 488 in the NG group, which may introduce additional bias or affect statistical power. Medications taken by patients, which were not included in the analysis, represent another source of confounding, as some medications may contribute to weight gain. The absence of this information prevents a comprehensive assessment of the relationship between diet quality, medication use, and the health conditions under study, thereby limiting the ability to draw robust conclusions regarding the impact of nutritional habits and pharmacological factors on patient health. Hence, these intrinsic limitations should be carefully considered when extrapolating the study’s results to larger, more diverse populations or different clinical environments, particularly in contexts where dietary factors and medication use play a critical role in patient health and treatment outcomes.

### 2.8. Study Strengths

This study stands as a pioneering effort to evaluate the impact of pregnancy on WLT following bariatric surgery and to explore the relationship between patient age and initial weight. It contributes to a deeper understanding of post-operative outcomes in a patient population that has not been extensively studied before. The findings have the potential to guide clinical decisions and patient management in a domain where evidence is currently limited.

## 3. Results

A total of 508 patients were included in this retrospective study. Overall, 20 patients (3.9%) were part of the GG, and 488 patients (96.1%) were part of the NG. The most common comorbidity in the NG group was hyperlipidemia (41.3%), and in the GG, it was GERD (30.0%). In NG, the prevalence rates for hypertension, hyperlipidemia, diabetes mellitus (DM), and obstructive sleep apnea (OSA) were 30.7%, 41.3%, 26.8%, and 23.5%, respectively. In contrast, GG had lower prevalence rates with hypertension at 10.0%, hyperlipidemia at 25.0%, DM at 10.0%, and OSA at 5.0%. There were no smokers within 1 year before the surgery in the GG, but the NG reported 3.0% of patients who were smokers. There were more insulin users in the NG (10.8%) compared to the GG (0%). On the contrary, orlistat use and anticoagulation use were more common in the GG (55.0% and 5.0%, respectively) compared to the NG (28.8% and 2.8%, respectively).

The analysis of the American Society of Anesthesiologists (ASA) class distribution among patients indicated that ASA III was the most prevalent category in both the non-gravid group (70.0%) and the gravid group (63.9%). ASA II was also prevalent, accounting for 34.2% in the non-gravid group and 30.0% in the gravid group. ASA I and ASA IV were less frequently observed in both groups (Table 1).

The mean initial BMI for GG and NG was 47 kg/m^2^ and 43 kg/m^2^, respectively. During PoPG, adjusted weight in both groups follows the path across time (Table 2, P1). For GG, weight decreases initially in PoWG (Table 2, P2), with the highest difference in weight between GG and NG being 2.913 kg on day 339. Then, the weight increases for GG during PoWG (Table 2, P2), with the highest difference between the two groups being 5.210 kg on day 498. For GG during PoPP, weight immediately decreases after delivery, with the maximum difference being 3.963 kg between the two groups, and then increases over time to levels similar to NG (Table 2, P3). Weight differences 6 months PoPP for GG and NG were not statistically different. Older age was associated with reduced weight loss during PoPG (Table 2, M, days post-op (linear) by Baseline Age), while higher initial weight was associated with increased weight loss during PoPG (Table 2, M, days post-op (linear) by Baseline Weight). In both instances, these effects attenuate over time (Table 2, M, age and weight quadratic terms).

## 4. Discussion

This study found that the changes in weight are similar between GG and NG before pregnancy. During pregnancy, the WLT decreased for GG and then increased compared to NG. In the post-partum period, the weight loss for GG had a sharp decline and then increased to a level similar to NG and then continued to follow the same path as shown in (Figure 2). Furthermore, this study also showed that older age or lower initial weight was associated with less weight loss during the period after surgery and before pregnancy (PoPG).

The age of a patient is an important factor for medical interventions, including bariatric procedures [16]. The association between age and weight loss after bariatric surgery can be attributed to different energy requirements, as well as psychopathological and behavioral hypotheses [16]. Toth et al. reported that aging is linked to reduced lipolytic capacity, which can lead to adipose tissue accumulation in older patients [16,17,18]. Moreover, total energy expenditure decreases with age, and the presence of sarcopenia further impedes weight loss [16,19]. A cross-sectional study by Contreras et al. involving 337 patients undergoing bariatric surgery, with 72.5% being female, reported patients younger than 45 years old significantly had a higher percentage of excess BMI loss compared to older patients [19]. Similarly, Woźniewska et al. also reported a significantly higher percentage of excess BMI loss in patients < 45 years old in a retrospective study of 555 patients [16]. Our study’s findings also demonstrate older age is associated with reduced weight loss. Furthermore, higher initial weight could lead to greater weight loss compared to lower initial weight, which may be explained by the increased energy cost of weight bearing activities such as walking and standing related to greater body weight.

Weight gain during pregnancy could have a direct impact on both the immediate and future maternal and fetal health and may also contribute to the development of multisystemic disorders [20]. The National Academy of Medicine (NAM) provided gestational weight gain recommendations based on maternal pre-pregnancy BMI [4,21]. Gestational weight gain below the lower limit of 5 kg in women who are overweight or women with obesity is associated with an increased risk of small for gestational age (SGA) neonates, as well as decreased neonatal fat mass, lean mass, birth length, and head circumference [4,13,22]. Heusschen et al. reported significantly lower gestational weight gain (below the NAM recommendation) for pregnancies within 12 months after surgery compared to pregnancies after 12 months when studying 196 singleton pregnancies [4]. Consequently, the authors reported significantly lower neonatal birth weight for pregnancies within 12 months. However, the authors did not report any significant difference in the risk for SGA neonates, nor was there any relation between pregnancy-related complications and surgery-to-conception interval or gestational weight gain. The WLT in our study shows that there is a maximum increase in weight by >5 kg in the third trimester, which is above the NAM recommendation.

It is crucial for healthcare providers to be knowledgeable of the various types of bariatric surgery in order to monitor for possible nutritional deficiency sequelae and proactively manage the impact of altered micronutrient absorption. Malabsorptive procedures such as RYGB and biliopancreatic diversion (BPD) bypass modify the tract of the small intestine and reduce absorption of micronutrients, whereas restrictive procedures such as laparoscopic adjustable gastric banding (LAGB) and SG decrease stomach capacity [23]. While there have been case reports of congenital anomalies occurring after malabsorptive procedures due to maternal malnutrition, the evidence from observational studies and clinical trials remains limited [23,24]. Previous reports demonstrate that iron absorption and vitamin B12 deficiency worsen during pregnancy, especially following bariatric surgery, resulting in significant iron deficiency anemia (IDA) and vitamin B12 issues. This condition is frequently encountered in clinical settings [24,25].

Post-bariatric surgery pregnancy complications also vary by procedure type. Pregnancies following RYGB have been associated with an incidence as high as 8% of internal hernia [6,26,27]. The most common symptoms of internal hernia include upper abdominal pain, nausea, and vomiting, which can be easily mistaken for early pregnancy symptoms and therefore ignored [6,28,29,30]. The symptoms of band slippage can also be mistaken for early pregnancy symptoms [30]. Additionally, the psychological impact of gestational weight gain should not be underestimated. Many women fear gaining weight during pregnancy, and healthcare professionals should be aware of the underlying factors, encourage adequate weight gain for a healthy pregnancy [4], and counsel the patient on reassuring post-bariatric pregnancy WLTs.

In addition to the benefits of pregnancy after bariatric surgery discussed earlier, this study demonstrates that the change in weight eventually returns to a trend similar to non-gravid individuals after delivery. Thus, patients can be reassured that the outcomes of bariatric surgery remain safe with appropriate monitoring [6]. Women of childbearing age who plan to undergo or have undergone bariatric surgery should be provided preconception counselling [6]. Counselling should include patient education on the benefits and risks of pregnancy post-surgery, concerning symptoms, potential nutritional deficiencies, nutritional guidance (including referral to an experienced nutritionist with bariatric knowledge), psychological support, multidisciplinary bariatric team follow-up, and disclosure to her pregnancy provider of the bariatric surgery history [6].

A few studies have looked at the relationship between gestational weight gain and pregnancy outcomes [4,13,14,15]. However, to our knowledge, this study is the first to examine weight loss trajectories (WLTs) following bariatric surgery before, during and after pregnancy. There were several limitations of this study: 1. the impact of the COVID-19 pandemic during the follow-up period could have affected data collection and outcomes; 2. as a retrospective study, there is a possibility of selection and/or information bias; 3. the number of patients in GG was limited and lower than in NG; and 4. there is a lack of information regarding breastfeeding, which could have impacted weight changes and maternal health.

## 5. Conclusions

In conclusion, this model suggests that pregnancy after bariatric surgery affects WLTs during PoWG and PoPP, and no difference in weight loss is expected 6 months post-pregnancy. Older age and lower initial weight were associated with decreased weight loss during the PoPG phase, and hence, age and initial weight could be considered prognostic factors. Patients wishing to conceive should be advised to avoid pregnancy during the period of rapid weight loss and should be informed that WLTs may vary during pregnancy and early post-partum. Overall, this study provides a better understanding on the weight loss dynamics before, during and after pregnancy following bariatric surgery.

## Figures and Tables

**Table 1:** Type of surgery and number of patients with pre-existing comorbidities or on medication.

	NG = 488	GG = 20
Age (years), mean	37	33
Primary procedure, no. (%)		
RYGB	227 (46.5)	10 (50.0)
SG	261 (53.5)	10 (50.0)
Pre-existing comorbidities, no. (%)		
Hypertension	150 (30.7)	2 (10.0)
Hyperlipidemia	202 (41.3)	5 (25.0)
DM	131 (26.8)	2 (10.0)
GERD	149 (30.5)	6 (30.0)
OSA	115 (23.5)	1 (5.0)
Smoker within 1 year before surgery	15 (3.0)	0
Medication use, no. (%)		
Insulin	53 (10.8)	0
DM	152 (31.1)	5 (25.0)
Orlistat	141 (28.8)	11 (55.0)
Steroid/Immunosuppressant	16 (3.2)	0
Anticoagulation	14 (2.8)	1 (5.0)
ASA status		
ASA I	9 (1.8)	0
ASA II	166 (34.2)	6 (30.0)
ASA III	311 (63.9)	14 (70.0)
ASA IV	2 (0.4)	0

Abbreviations: diabetes mellitus (DM), gastroesophageal reflux disease (GERD), obstructive sleep apnea (OSA), and American Society of Anesthesiologists (ASA) class.

**Table 2:** WLT: mixed-effects results.

Model Section Code	Model Section Name (Group)		Trajectory Parameter Name	Estimate	Std. Error	df	*t*	*p*	95% CI of Estimate	
									Min	Max
P1	Phase 1 (post-surgery/pre-gravid)	Common	Wt at Day 0 (Intercept)	105.396	0.199	723.235	530.785	<0.001	105.007	105.786
			Days post-op (linear)	−0.129	0.001	6626.618	−127.108	<0.001	−0.131	−0.127
			Days post-op (quadratic)	1.65 × 10^−4^	2.10 × 10^−6^	6566.227	78.753	<0.001	1.61 × 10^−4^	1.69 × 10^−4^
			Days post-op (cubic)	−5.97 × 10^−8^	1.10 × 10^−9^	6540.807	−54.491	<0.001	−6.19 × 10^−8^	−5.76 × 10^−8^
P2	Phase 2 (post-surgery while gravid)	GG	Days Gravid (linear)	-0.126	0.043	6649.979	−2.944	0.003	−0.210	−0.042
			Days Gravid (quadtratic)	0.002	0.001	6562.778	2.837	0.005	4.81 × 10^−4^	0.003
			Days Gravid (cubic)	−3.99 × 10^−6^	1.64 × 10^−6^	6533.897	−2.428	0.015	−7.22 × 10^−6^	−7.70 × 10^−7^
P3	Phase 3 (Post-partum)	GG	Wt change at Post-partum (Intercept)	−6.386	4.121	6511.170	−1.550	0.121	−14.463	1.692
			Days Post-partum (linear)	0.170	0.099	6518.712	1.716	0.086	−0.024	0.365
			Days Post-partum (quadratic)	0.001	0.001	6510.628	1.873	0.061	−6.47 × 10^−5^	0.003
			Days Post-partum (cubic)	4.08 × 10^−6^	1.65 × 10^−6^	6534.923	2.476	0.013	8.50 × 10^−7^	7.30 × 10^−6^
M	Phase 1 Trajectory Controls/Moderators	Baseline Age	Wt at Day 0 (Intercept) by Baseline Age	0.031	0.016	657.887	1.932	0.054	0.000	0.063
			Days post-op (linear) by Baseline Age	0.001	4.67 × 10^−5^	6661.029	17.322	<0.001	0.001	0.001
			Days post-op (quadratic) by Baseline Age	−5.59 × 10^−7^	4.63 × 10^−8^	6586.543	−12.068	<0.001	−6.50 × 10^−7^	−4.69 × 10^−7^
		Baseline Weight	Wt at Day 0 (Intercept) by Baseline Weight	0.962	0.011	647.513	89.521	<0.001	0.941	0.983
			Days post-op (linear) by Baseline Weight	−0.001	2.83 × 10^−5^	6647.054	−24.113	<0.001	−0.001	−6.26 × 10^−4^
			Days post-op (quadratic) by Baseline Weight	4.70 × 10^−7^	2.54 × 10^−8^	6592.478	18.471	<0.001	4.20 × 10^−7^	5.19 × 10^−7^

## Data Availability

The data presented in this study are not available due to privacy.
